# Does Early Treatment Prevent Deafness in Thiamine-Responsive Megaloblastic Anaemia Syndrome?

**DOI:** 10.4274/jcrpe.v3i1.08

**Published:** 2011-02-23

**Authors:** Leyla Akın, Selim Kurtoğlu, Mustafa Kendirci, Mustafa Ali Akın, Musa Karakükçü

**Affiliations:** 1 Department of Pediatrics, Erciyes University Faculty of Medicine, Kayseri, Turkey; +90 533 240 16 43 leylabakin@gmail.comErciyes University, Faculty of Medicine, Department of Paediatrics, 38039, Kayseri, Turkey

**Keywords:** Thiamine-responsive megaloblastic anaemia, Diabetes, deafness

## Abstract

Thiamine-responsive megaloblastic anaemia (TRMA; OMIM 249270) syndrome is an autosomal recessive disorder characterized by diabetes mellitus, megaloblastic anaemia, and sensorineural deafness. Progressive hearing loss is one of the cardinal findings of the syndrome and is known to be irreversible. Whether the deafness in TRMA syndrome can be prevented is not yet known.  Here, we report a four-month-old female infant diagnosed with TRMA syndrome at an early age. There was no hearing loss at the time of diagnosis. The patient’s initial auditory evoked brainstem response measurements were normal. Although she was given thiamine supplementation regularly following the diagnosis, the patient developed moderate sensorineural hearing loss at 20 months of age, indicating that early diagnosis and treatment with oral thiamine (100 mg/day) could not prevent deafness in TRMA syndrome. It would be premature to draw general conclusions from one case, but we believe that further patient-based observations can shed light on the pathophysiology of this rare syndrome as well as prediction of its prognosis.

**Conflict of interest:**None declared.

## INTRODUCTION

Thiamine-responsive megaloblastic anaemia (TRMA; OMIM 249270) syndrome is an autosomal recessive disorder characterized by diabetes mellitus, megaloblastic anaemia, and sensorineural deafness. The syndrome was first described by Porter et al (1) . Thirty years later, SLC19A2, the gene encoding a high-affinity thiamine transporter protein (THTR-1), was identified by three independent groups (2,3,4). To date, 29 distinct mutations in this gene have been described in a total of 70 reported patients (5). Progressive hearing loss is one of the cardinal findings of the syndrome and is known to be irreversible. However, most of the TRMA cases reported to date have been diagnosed after infancy and the hearing loss was already present in many at the time of diagnosis. Here, we report a four-month-old female infant who presented with megaloblastic anaemia and hyperglycemia and was diagnosed as a case of TRMA syndrome in the absence of hearing loss. Although treatment with thiamine was started at an early age, development of deafness at 20 months of age could not be prevented. 

## CASE REPORTS

A four-month-old girl presented to our clinic with vomiting and pallor that was noticed about one month ago. She was born to nonconsanguineous parents at term after an uneventful pregnancy with a birth weight of 3800 g. She was the fourth child of the parents. The infant was breast-fed from birth onwards. Family history was negative for diabetes, anaemia or deafness. On admission, the patient’s weight was 5800 g (25^th^ percentile), height was 60 cm(25^th^ percentile), and head circumference was 39.5 cm (25^th^ percentile). Physical examination  findings were normal, except for pallor. Audiological and ophthalmological examinations were also normal. Laboratory investigations showed anaemia, thrombocytopenia and hyperglycemia. White blood cell count was 7 000/μL with 70% lymphocytes. Other haematological findings: Hb 6.6 g/dL, Hct 20.6%, MCV 96.3 fL, platelets 50 000/μL. Reticulocyte count was 1%.  Blood smear revealed anisocytosis, poikilocytosis, and macrocytic erythrocytes along with some polynucleated cells. Biochemical findings were: glucose  280 mg/dL, ALT 25 U/L, AST 35 U/L, BUN 10 mg/dL, creatinine 0.5 mg/dL, Ca 9.0 mg/dL, P 4 mg/dL, Na 132 mmol/L, and K 5 mmol/L. Blood gas analysis was normal. On follow-up, blood glucose levels were found to show small changes, around 200 mg/dL. HbA1c level was 5% (N: 3.5-6.5), while insulin and C-peptide levels were 0.9 mIU/mL (N: 4-16) and 0.05 ng/mL (N: 0.20-0.52), respectively. Folic acid and vitamin B12 levels were normal being 7.25 ng/mL (N: 3-17) and 626 pg/mL (N: 193-985), respectively. Vitamin B1 level was 13.5 μg/L (N: 28-85).

Examination of the bone marrow aspirate specimen demonstrated increased megaloblastic erythropoiesis and megakaryopoiesis.

The otoacoustic emission test, which was performed at neonatal screening for deafness, was normal. The repeated test as auditory evoked brainstem responses (ABRs) at the onset of the disease was also normal. 

The findings of severe macrocytic anaemia, thrombocytopenia and hyperglycemia led us to consider a presumptive diagnosis of TRMA, and oral thiamine therapy in a dose of 100 mg/day was started. Hyperglycemia resolved three days later. Haemoglobin levels increased gradually and reached 10.6 g/dL by day 16 of the treatment. Platelet count also increased and normalized within 5 days. The diagnosis of TRMA was confirmed by molecular genetic testing, which showed a homozygous mutation in SLC19A2 gene with two-base-pair deletion and three-base insertion, 566_567delGCinsTCT, that results in Insdel 189fs/ter239.

At the follow-up visit at age 20 months, the patient, who had been receiving 100 mg/day of oral thiamine supplementation, had neither anaemia nor hyperglycemia. Her somatic and motor development was appropriate for age, but she could utter only a few words. The parents were not aware of any hearing loss. ABR test was performed and bilateral moderate sensorineural hearing loss was detected. The patient was referred to an otolaryngologist for evaluation for hearing device implantation.

## DISCUSSION

Thiamine pyrophosphate, also known as vitamin B1, is a coenzyme required for several key steps in intermediary metabolism (6). The deficiency of SLC19A2 gene product THTR-1 results in defective transport of thiamine into cells (7).

TRMA syndrome is an autosomal recessive disorder characterized by diabetes mellitus, megaloblastic anaemia, and sensorineural deafness. In addition to these cardinal components, other findings including thrombocytopenia, pancytopenia, optic atrophy, retinal degeneration, situs inversus, cardiomyopathy, arrhythmias, congenital heart defects, and stroke have been reported in association with TRMA syndrome (8,9,10,11). In our patient, megaloblastic anaemia was accompanied by thrombocytopenia and both responded well to the treatment with thiamine within two weeks. Cardiological and ophthalmological examinations revealed no pathology. The gene SCL19A2 causing TRMA syndrome has been mapped to chromosome 1q23.2-23.3 (12). To date, 29 distinct clinical mutations have been 

identified in SLC19A2 comprising point mutations, as well as premature truncations resulting from missense, nonsense, frameshift, and very recently, from compound heterozygous mutations (5). We found a homozygous mutation with two-base-pair deletion and three-base insertion, 566_567delGCinsTCT, which results in Insdel 189fs/ter239. It is predicted to be null. The mutation found in this case has been recently described as a novel mutation in another Turkish family unrelated to our patient and living in a different geographical area. That patient was diagnosed at age two years and, contrary to our case, had already developed deafness (13). We suggest that a not-yet-determined founder effect for this allele may be responsible for the difference in the timing of development of deafness.  

Diabetes mellitus in TRMA syndrome is due to a nonimmune mechanism and is most likely secondary to impairment of islet cell function caused by intracellular 

thiamine deficiency. In this syndrome, diabetes usually develops in early childhood and the response to thiamine treatment is variable (14). At puberty, probably due to increased b-cell apoptosis, insulin may be required in addition to thiamine treatment to control diabetes (15). Although diabetic ketoacidosis is rare in TRMA patients, it can occur even in prepubertal ages if thiamine supplementation is insufficient (16). Although the initial levels of serum insulin and C-peptide were low in our case, hyperglycemia resolved in three days by thiamine treatment only. Insulin was not required and hyperglycemia did not recur during the follow-up period of about two years.

Sensorineural deafness is one of the cardinal findings of TRMA syndrome. A high-affinity transporter protein is expressed in the inner hair cells within the cochlea (17). To further study the disease, a mouse model of TRMA was created with targeted disruption of SCL19A2 gene (18). In their study describing auditory phenotype of this mouse line, Liberman et al (19) demonstrated selective loss of inner hair cells after 1-2 weeks on low-thiamine diet, which is an uncommon pattern for sensorineural hearing loss. Obviously, the degree of inner hair cell loss depended on the duration and magnitude of thiamine deprivation. Interestingly, they showed normal cochlear function in these mutants when maintained on a high-thiamine diet. In another animal study, Oishi et al (20) showed that ABR thresholds were markedly high in SCL19A2 (-/-) mice on thiamine-free diet, while normal in thiamine-fed SCL19A2 (-/-) mice and in the wild types. These findings may support the hypothesis that early treatment with thiamine can prevent hearing loss in patients with SLC19A2 gene mutations. Initially, we speculated that the hearing loss in the present case might have been prevented by early treatment. Unfortunately, despite the regular thiamine supplementation (100 mg/day), deafness developed at age 20 months. 

Since patients usually present to a clinic at a late stage, it is difficult to define the actual time of symptom onset. Mild hearing loss may not be noticed by parents in the early months of life, therefore, all infants should be evaluated by hearing tests. In our patient, the initial otoacoustic emission test, which was performed at neonatal screening for deafness, was normal. The ABRs measured at age four months were also normal. However, the repeated test (ABR) performed at 20 months of age, when the patient was under regular thiamine treatment, showed bilateral moderate sensorineural hearing loss. Long-term follow-up findings of three patients with TRMA have been reported recently (21). Two of these patients were already deaf on presentation, but the youngest one was symptom-free till hyperglycemia developed at age 3. Although thiamine treatment was 

started at that age, hearing loss occurred at age 5. 

In prior reports, all patients with mutations in SLC19A2 were homozygous. In a very recent paper, Bergmann et al (5) reported for the first time four compound heterozygous mutations. Interestingly, two of these patients possessed less severe hearing phenotype-one had normal hearing even at age 15 and the other one had only minimal hearing loss at age 30. It was not clarified whether these milder phenotypes were attributable to compound heterozygosity. Our patient had a known homozygous mutation in SLC19A2. To our knowledge, there is no case report published in the literature with such mild hearing phenotype among TRMA patients with homozygous mutations.

Recently, a one-month-old female patient with TRMA syndrome who presented without diabetes or hearing loss and did not develop deafness until 32 months of age was reported from Turkey (22). The mutation identified in that case, 242insA, was different from the one in our patient. The authors suggest that deafness was prevented by early diagnosis and treatment, but we believe further follow-up is needed to confirm this conclusion. 

The dose of thiamine necessary to treat or prevent the symptoms of TRMA syndrome and the timing of therapy are not yet clarified. The time of onset of histopathological changes caused by intracellular thiamine deprivation is also not known. Theoretically, these alterations may begin even in intrauterine life. However, it seems logical to assume that the earlier the treatment starts, the better the response will be in these patients. 

In conclusion, TRMA syndrome should be kept in mind in the differential diagnosis of hyperglycemia coexistent with megaloblastic anaemia. Deafness, a cardinal component of the syndrome, may not be present at the onset of the disease in infancy. Early institution of   appropriate thiamine supplementation does not appear to prevent hearing loss in these patients. However, it still needs to be proven whether early treatment with higher doses of thiamine can prevent deafness. As the number of cases diagnosed with TRMA syndrome at a very early age increases, the role of thiamine in prevention of deafness will be further elucidated by future studies.

## ACKNOWLEDGEMENT

The authors are grateful to Mr Neufeld (Division of Hematology, Children’s Hospital, Boston, MA) for performing genetic testing of the patient. 
